# Increased [CO_2_] Causes Changes in Physiological and Genetic Responses in C_4_ Crops: A Brief Review

**DOI:** 10.3390/plants9111567

**Published:** 2020-11-13

**Authors:** Renan Gonçalves da Silva, Rita de Cássia Alves, Sonia Marli Zingaretti

**Affiliations:** 1 School of Agricultural and Veterinarian Sciences Jaboticabal, São Paulo State University (Unesp), Jaboticabal, 14884-900 São Paulo, Brazil; biotek_rere@hotmail.com; 2 Semi-Arid National Institute (INSA), Crop Production Center, Campina Grande, 58434-700 Paraíba, Brazil; rita.alves@insa.gov.br; 3 Biotechnology Unit, University of Ribeirão Preto (UNAERP), Ribeirão Preto, 14096-900 São Paulo, Brazil

**Keywords:** CO_2_ enrichment, C_4_ plants, gene expression, photosynthesis, productivity

## Abstract

Climate change not only worries government representatives and organizations, but also attracts the attention of the scientific community in different contexts. In agriculture specifically, the cultivation and productivity of crops such as sugarcane, maize, and sorghum are influenced by several environmental factors. The effects of high atmospheric concentration of carbon dioxide ([CO_2_]) have been the subject of research investigating the growth and development of C_4_ plants. Therefore, this brief review presents some of the physiological and genetic changes in economically important C_4_ plants following exposure periods of increased [CO_2_] levels. In the short term, with high [CO_2_], C_4_ plants change photosynthetic metabolism and carbohydrate production. The photosynthetic apparatus is initially improved, and some responses, such as stomatal conductance and transpiration rate, are normally maintained throughout the exposure. Protein-encoding genes related to photosynthesis, such as the enzyme phosphoenolpyruvate carboxylase, to sucrose accumulation and to biomass growth and are differentially regulated by [CO_2_] increase and can variably participate owing to the C_4_ species and/or other internal and external factors interfering in plant development. Despite the consensus among some studies, mainly on physiological changes, further studies are still necessary to identify the molecular mechanisms modulated under this condition. In addition, considering future scenarios, the combined effects of high environmental and [CO_2_] stresses need to be investigated so that the responses of maize, sugarcane, and sorghum are better understood.

## 1. Introduction

The increased atmospheric concentration of carbon dioxide ([CO_2_]) is one of the factors responsible for global warming [1] and the consequent irreversible climate change for hundreds of years [2]. This increase has been significant since the industrial revolution, and over the last 50 years, [CO_2_] levels have increased from 320 to 390 ppmv (parts per million volume) [3,4]. In 2018, [CO_2_] levels reached 409.23 ppmv [5] and there are some predictions that for 2100, [CO_2_] will reach 800 ppmv [4].

High [CO_2_], as well as radiation, temperature, and water availability, influence crops [6]. Climate change affects plant growth and development, mainly owing to changes in photosynthetic carbon assimilation [7]. Plants absorb atmospheric CO_2_ by chemically reducing carbon, and this reduction is actually much more, because in addition to storing chemical energy in the plant, photosynthesis also provides carbon skeletons for the organic molecules that comprise the vegetal structure [8]. Theoretically, plants can mitigate climate change through the photosynthetic conversion of atmospheric CO_2_ into carbohydrates and other organic compounds [9]. However, the direct and indirect effects of these changes on plants need to be better evaluated, especially regarding to their impact on crop production and productivity [7]. Increased [CO_2_] levels affect most physiological processes in plants; however, plants under this condition can present a mix of positive and negative responses [5].

C_3_ and C_4_ species respond differently to this increase [10] because [CO_2_] levels affect biological processes at different levels of organization [11]. There are many studies on plant responses to increased [CO_2_] with C_3_ species. Less attention has been given to C_4_ plants, owing to the assumption that the mechanism of CO_2_ accumulation in C_4_ plants makes them insensitive to high [CO_2_] levels [7].

However, studies on plants such as *Saccharum officinarum* L. [12,13] and *Sorghum bicolor* L. [14] in high-level CO_2_ environmental conditions indicate increased photosynthetic rates. Changes in the transpiration rate and metabolite production (e.g., glucose, mannose, and galactose) have been reported in *Zea mays* L. [9]. In addition, increased [CO_2_] can also improve plant susceptibility to pests and diseases, mainly because of decreased phytochemical and phytohormone production, with these being part of the plant defensive system [15,16].

Several genes were identified in C_3_ and C_4_ plants that are directly involved in plant responses to environmental conditions [17]. In soybeans (*Glycine max* ‘93B15’), 327 genes were responsive to increased [CO_2_], indicating that this environmental condition stimulates carbohydrate degradation, increasing energy and leaf expansion and growth precursors [18]. C_4_ plants under increased [CO_2_] presented changes in the expression of genes involved in the photosynthetic mechanism of *Zea mays* [19] and differential expression of dozens of genes also related to photosynthesis and development in sugarcane plants [13].

Environmental factors, such as water availability and temperature, are associated with a considerably increased atmospheric [CO_2_]. In C_3_ plants, the interactive effects of water deficiency and increased [CO_2_] were studied by Medina et al. [20] in wheat genotypes (*Triticum turgidum* L.), finding that these effects depend on genotype and severity of water stress, especially for the expression of genes responsive to water conditions, such as catalase and superoxide dismutase, negatively regulated in three of the four evaluated genotypes. More than 1600 differentially expressed genes involved in photosynthetic processes and stress response mechanisms were identified in sugarcane under water deficiency [21]. Molecular studies using cDNA arrays identified several genes induced by biotic and abiotic stresses [22,23], revealing that drought-tolerant or sensitive genotypes respond differently to this stress at the gene expression level [23].

Therefore, expanding our knowledge on physiological and genetic responses in different crops can improve the development of cultivars that are better adapted to the new climatic conditions to which they will be subjected in the coming decades. Considering the interest in elucidating these responses in C_4_ plants (sugarcane, maize, and sorghum), this brief review approaches the main effects of high [CO_2_] through considering different exposure periods (time) and emphasizing mostly physiological aspects related to photosynthetic metabolism and differential gene expression, despite the lack of data in the literature.

## 2. C_4_ Plants of Commercial Importance

Although C_4_ plants represent a small portion of the world’s plant species, they have substantial ecological and economic significance [12]. *Zea mays*, *Sorghum bicolor*, and *Saccharum* spp. stand out among C_4_ species and are considered the most important world crops in terms of production [24,25,26,27]. These plants are grasses belonging to the Poaceae family, generally growing in warmer climates with considerable light intensity [28,29].

Grains and cereals, such as *Zea mays* and *Sorghum bicolor*, are essential food group in the human diet, as they provide energy and essential nutrients. They are also a source of food for animals and of raw materials to produce biocomposites. Maize is currently the most cultivated crop in the world, being used as food and raw material for ethanol, starch, and oil production [30]. However, climatic instability can influence crop yield, as is the case in the south of Brazil. Climate changes have negatively influenced the productive potential of maize crops (0.8% reduction in average productivity levels) [31]. Sorghum has also become important in terms of cultivation, being used in animal feed or as a food base for millions of people, mainly in Africa and Asia [32]. A considerable portion of the global sorghum harvest areas are in Africa and Asia. The majority of sorghum production is in Africa (41%), the Americas (38%), and then Asia (18%) [33]. Recent reports show growth in cereal production in the 2019/2020 harvest in Brazil, with an increase of 24.7% in sorghum production compared to the previous harvest.

*Saccharum* spp. is a grass of Asian origin that is known for its sucrose storage capacity in the stalks [34,35], and is considered a robust crop with efficient biomass production and great potential for bioenergy production [36]. Brazil has the largest production in the world, estimated at 35.3 million tons of sugar and 29.3 billion liters of ethanol in the 2019/2020 harvest, followed by India, China, and other countries [37].

The productivity of these crop plants can be negatively or positively influenced, mainly owing to cultivation in different regions and the consequent effects of atmospheric [CO_2_], global warming, and precipitation rate changes [38,39]. Recent projections show significantly decreased yields in major crops until the end of the 21st century, even with the effects of CO_2_ fertilization on crops [40,41]. Understanding plant responses to increased [CO_2_] is essential to predict net primary productivity and for ecosystem service provision, which can meet future food and fuel demands [41].

## 3. C_4_ Photosynthesis Mechanism and CO_2_

In plants, carbon is assimilated through the phosphate-reducing cycle (Calvin cycle) [25]. Once CO_2_ has been absorbed into the aerial space of the leaf and mesophyll, it is converted into bicarbonate and pre-assimilated into the mesophyll cytoplasm by the enzyme phosphoenolpyruvate carboxylase (PEPC) [42,43]. Some plants have a C_4_ biochemical mechanism, responsible for photosynthetic processes. Approximately 8100 plant species use C4 carbon fixation [44], and on the basis of the decarboxylation mechanism in the perivascular sheath, these plants can be classified into different subgroups [45]. Unlike the C_3_ photosynthetic pathway, which has only one carboxylation step, the C_4_ photosynthetic pathway has two carboxylation steps [46].

During photosynthesis, the incident light is absorbed by chlorophyll molecules, responsible for transferring the captured energy to photosystem I (PSI) and II (PSII) reactions, which direct the energy to the electron transport chain [47]. The CO_2_ absorbed by C_4_ species is diffused through the stomata and hydrated by the carbonic anhydrase enzyme [48], being subsequently assimilated by oxaloacetate using PEPC located in the cytosol as the substrate [49]. Upon receiving the electrons, oxaloacetate is reduced to malate or aspartate, or even both. It depends on the subtype of C_4_ species, as they are divided by the type of enzyme that performs the decarboxylation: nicotinamide adenine dinucleotide phosphate-malic enzyme (NADP-ME), nicotinamide adenine dinucleotide-malic enzyme (NAD-ME), and phosphoenolpyruvate carboxykinase (PEPCK) [50,51,52,53].

The CO_2_ is supplied to the active site of the enzyme ribulose-1,5-bisphosphate carboxylase-oxygenase (Rubisco), located in the chloroplasts of the vascular sheath cells, and is used for carbohydrate production [49,54]. According to Jenkins et al. [55], in C_4_ plants, [CO_2_] around the vascular sheath Rubisco is 10 times higher than the [CO_2_] present in the atmosphere, explaining the importance of this enzyme in the C_4_ photosynthetic apparatus. 

Plants with the C_4_ mechanism use these biochemical processes to concentrate CO_2_ in the leaves where the Rubisco enzyme acts by fixing CO_2_ [8]. As this is minimally affected by increased [CO_2_], this is probably directly related to the high [CO_2_] in the sheath cells of C_4_ plants. Increased [CO_2_] often has little direct effect on the photosynthetic rates of these plants; however, they reduce stomatal conductance, consequently improving photosynthesis [56,57].

The Rubisco enzyme changes the flow of atmospheric CO_2_ in carbon assimilation [58]. At current CO_2_ levels, this assimilation can be saturated in C_4_ species, mainly because PEPC uses HCO_3_ (bicarbonate system) as the substrate instead of CO_2_ [59]. Although studies that consider the [CO_2_] mechanism in C_4_ plants under high [CO_2_] conditions are less comprehensive, it is remarkable that global concerns regarding climate change and the real impacts on the productivity of several crops have led to studies that evaluated how these plants will respond to the predicted [CO_2_] increase. What are the effects on plant development and gene response modulation? Are the physiological mechanisms changed? These questions have been investigated through experiments evaluating high [CO_2_] application, experimental conditions, periods of stress exposure, and integrative stress effect (but little research involving genetic machinery), among other experimental aspects (Table 1).

Despite the limited number of published studies, they present a similar context, pointing to the same line of knowledge that, owing to their [CO_2_] mechanisms, C_4_ plants have an almost saturated photosynthetic capacity under the current atmospheric CO_2_ conditions. In this context, many questions arise, e.g., how do C_4_ plants really respond to high [CO_2_] conditions? Understanding how these plants, economically important for the world, are influenced by increased [CO_2_] levels is essential for the development of breeding programs aimed at improving or adapting plant growth and increasing productivity considering future climate changes [12].

## 4. Physiological, Biochemical, and Gene Expression Changes

Increased [CO_2_] levels affect morphological, physiological, and productive plant aspects [67]. Many growth parameters have been shown to be positively altered in this climatic condition. In maize at the vegetative stage, there is an increase in leaf area (over 50%) in 550 ppm [CO_2_] [68], and in studies with sugarcane (30%) [12] and sorghum (25%) [69], in 720 μmol mol^−1^ and 900 µL CO_2_ L^−1^, respectively. Other measurements, such as plant height and biomass are increased under high [CO_2_] (Table 2). According to Souza et al. [13] in sugarcane crops it was observed that productivity can increase in environments with high [CO_2_], however, just as the authors report, it is necessary to consider that some factors influence these growth responses (e.g., the experiment was not performed under field conditions).

Photosynthetic metabolism, plant growth, and biomass accumulation are directly related to productivity. Sorghum and maize showed no increase in productivity of grain yield under high [CO_2_] conditions [70,71], and in sugarcane, the productivity, evaluated by the results of direct allocation of sucrose to the stem, was satisfactory under high [CO_2_] conditions [13,72]. However, experimental and environmental factors can improve different responses, as observed for some growth characteristics (leaf area and biomass) in maize, resulting in improved crop yield under high [CO_2_] conditions associated with increased precipitation [73].

**Figure 1:**
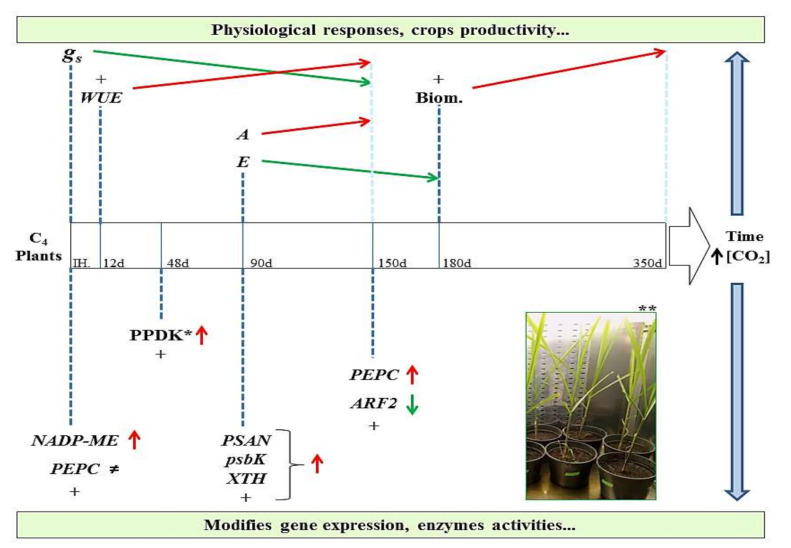
Schematic representation of some effects of elevated CO_2_ on C_4_ plants (maize, sugarcane, and sorghum). The green arrows indicate inhibition or decrease, while the red arrows indicate promotion or increase. The (+) sign indicates that other physiological parameters or genes/enzymes have also been modulated, and the (≠) sign indicates differential expression in some cultivars (plants C_4_) or experiments. * Represents pyruvate phosphate dikinase (PPDK) enzyme activity. ** Example of experiments under elevated CO_2_ conditions: sugarcane plants evaluated in fitotron plant growth chamber.

Some studies evaluated the physiological, biochemical, and molecular responses of C_4_ plants under high [CO_2_] conditions [12,13,19,62,63,64,75,77]. Figure 1 summarizes some of the main effects of high [CO_2_] on C_4_ plant photosynthesis, specifically maize, sugarcane, and sorghum. In this schematic representation, we emphasize physiological and genetic changes that occur owing to the duration of exposure of C_4_ plants to excess CO_2_.

Carbohydrates are the main products generated in the photosynthetic process, being processed in the stem and accumulated in the form of sugars in sugarcane [78]. Thus, increased photosynthetic rates and sucrose content indicate that the main regulation of sugarcane photosynthesis in high [CO_2_] [5] may occur through the light capture system, being possibly related to the increased electron rate of these plants and the expression of genes related to light capture and electron transport [13]. Several genes related to reserve accumulation and mobilization, cell cycle, and growth have been shown to be sensitive to changes in sugar concentrations [79], mainly sucrose [80]. Souza et al. [13] reported that among the main gene categories regulated in sugarcane after 150 days of exposure to elevated [CO_2_] are those related to the cell cycle and plant development, along with a significant difference in height and biomass between control and treated plants. The increased biomass of C_4_ plants under high [CO_2_] was typically attributed to reduced stomatal opening and conductance, as well as to subsequent improvement in the leaf water status [64,81] (Figure 1).

Sugarcane also shows a positive regulation of a few specific genes involved in sugar metabolism, such as the induction of the xyloglucan endotransglucosylase/hydrolase (XTH) gene after 90 days under high [CO_2_] [13]. XTH can hydrolyze xyloglucan backbones, consequently contributing to plant tissue expansion [82], thus suggesting a greater potential for leaf cells to expand (increased leaf area) and accumulate more sugars [13]. 

Genes related to PSI and PSII (photosystem I reaction center subunit N (PSAN) and photosystem II protein K (psbK)) were positively regulated in sugarcane after 90 days under high [CO_2_], concomitantly with the reported increase in CO_2_ assimilation rate [13]. In contrast, genes encoding Rubisco-related subunits were inhibited, as reported in maize by Huang et al. [62]. Therefore, despite the probable photosystem and electron transport chain functional normality in C_4_ plants due to increased atmospheric CO_2_, inhibited or lower CO_2_ fixation by Rubisco in the Calvin cycle may be possible. In addition, C_4_ plants invest more in the PEPC and pyruvate phosphate dikinase (PPDK) photosynthetic cycle and less in Rubisco [83].

Some factors, such as, the experimental conditions ([CO_2_] levels and exposure time) can influence in C_4_ plants responses. In general (but lacking consensus), it is understood that the photosynthetic apparatus may acclimatize in the initial periods of plant exposure to high [CO_2_] [83]. In relation to control plants (ambient [CO_2_]), it has been shown that CO_2_ assimilation increases (A) [13], which was also observed in the increasing exchange rate of leaf CO_2_ in sorghum, maize, and sugarcane, especially in younger leaves [12,84,85]. Leaf CO_2_ exchange rate increase (20%) in sugarcane plants (7 days after leaf emergence) grown at double-ambient (720 µmol mol^−1^) CO_2_ [12].

Photosynthesis of the sorghum second leaves of FACE-grown plants (6 days, under daytime values of 566 µL l^−1^ and 373 µL l^−1^ [CO_2_] and the mean nighttime values of 607 µL l^−1^ and 433 µL l^−1^ [CO_2_] for FACE and control, respectively) was 37% greater than that of control-grown plants [84], and in youngest fully expanded leaf of maize grown under 550 µmol mol^−1^ [CO_2_], authors observed at times that A was up to 41% greater in leaves grown and measured under elevated [CO_2_] compared with ambient [CO_2_] [85]. Similar effects of increased [CO_2_] to other physiological responses in relation ambient [CO_2_] (control plants) have been shown in these three C_4_ species. The stomatal conductance (gs) decreases [13,19,64]; the transpiration rate (E) decreases [64,77]; and the expression of PEPC, NADP-ME, and other genes/enzymes related to photosynthetic metabolism changes [13,19] (Figure 1).

Some responses remain unchanged (for example, gs) with the long-term exposure of sugarcane plants (70–120 days) to high [CO_2_] (720 ppm), but other processes can be adjusted (regulated), such as the decreased photosynthetic capacity of plants due to carbohydrate accumulation in the leaf [13,62,86] and the modulation of the expression levels of genes related to growth, such as auxin response factor 2 (ARF2). Some studies showed the essential roles of ARFs in plant growth and development, as well as in adapting to stress [87,88]. However, the role of ARF2 becomes evident in the results reported by Lim et al. [89] as a true repressor of auxin signaling, that is, it directly interferes with the coordination of plant growth [90]. The repression of ARF2 in sugarcane after 150 days of exposure to 720 ppm [CO_2_], the differential expression of carbohydrate biosynthesis-related genes in sugarcane, such as glucose-6-phosphate dehydrogenase and xyloglucan endo-transglycosylase/hydrolase [13], and in maize exposure to 550 ppm [CO_2_], such as fructose-bisphosphate aldolase and glucose-1-phosphate adenylyltransferase [62], may explain the probable regulation of mechanisms related to growth control and the consequent biomass accumulation in C_4_ plants.

More experimental evaluations under high [CO_2_] are necessary to further understand the connection between photosynthetic capacity and the expression of many genes, as verified for the ribulose bisphosphate carboxylase small chain gene (rbcS) by Huang et al. [62]. In maize grown under increased atmospheric CO_2_, Rubisco, PEPC, NADP-MDH (NADP-malate dependent dehydrogenase), NADP-ME, and PPDK activities were negatively regulated [83], as also reported by Vu and Allen [63] in mature sugarcane (*Saccharum officinarum* L. ‘CP72-2086’) leaves. However, Rubisco, PPDK, and NADP-MDH enzymes were positively regulated in another sugarcane genotype under the same [CO_2_] conditions (*Saccharum officinarum* L. ‘CP73-1547’) [12].

C_4_ plants may show biochemical variations related to the type of decarboxylation enzyme [53] and probably in the role played by Rubisco in high atmospheric CO_2_ conditions. In maize, the expression of the NADP-ME gene was positively regulated in the B106 genotype, and the PEPC and NADP-MDH genes were negatively regulated in the same genotype. Different responses (except for the NADP-ME gene) were obtained in the analysis of the B76 maize genotype [19]. Therefore, genetic variability considerably influences different physiological and gene expression responses to high [CO_2_].

Little information is available about the molecular responses regulated in maize, sugarcane, and sorghum, and this approach shows the importance of C_4_ plants under high [CO_2_] conditions and the need of further studies involving this subject. The understanding needs to include aspects of physiology, metabolic mechanisms, and molecular mechanisms regulated in this condition, especially with regard to the photosynthetic enzyme efficiency, osmotic adjustment, antioxidant metabolism, and secondary metabolite production, among other processes that may directly or indirectly influence the adaptability responses to the climatic condition.

## 5. Perspectives

As previously reported, the elevated [CO_2_] contributes to the increase of biomass in maize, sugarcane, and sorghum. However, high [CO_2_] levels are closely related to water stress, one of the major factors of crop production and productivity losses [5]. How would a C_4_ plant sensitive to water stress react in conditions of high atmospheric [CO_2_]? Would it activate the same set of genes already identified under normal CO_2_ conditions? What are the physiological mechanisms and gene and/or protein profiles regulated under these conditions? 

**Figure 2:**
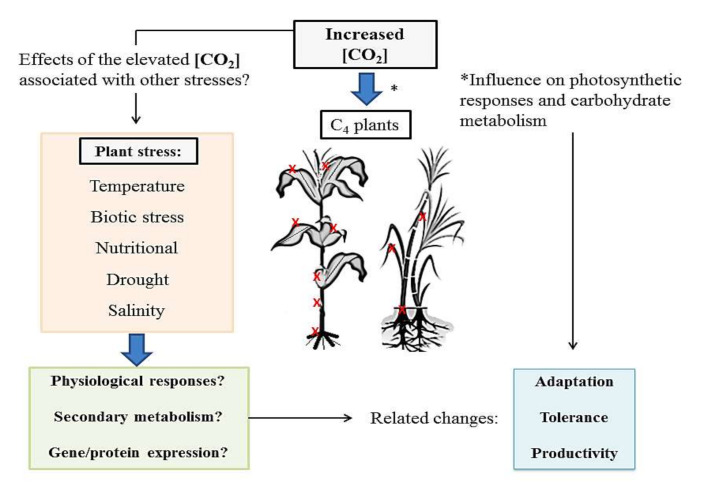
Environmental factors and increased [CO_2_] influence on physiology and general metabolism of C_4_ plants.

Increased [CO_2_] influences the photosynthetic and carbohydrate metabolisms. However, we know that the study efforts will be directed towards the investigation of the combined action of different environmental stresses on plants and high [CO_2_] (Figure 2). What is expected for the coming years are studies that involve physiological responses, secondary metabolism pathways, hormones, and mainly gene and protein profiles modulated by the combined effect, aiming at C_4_ plants more adapted to climatic diversities that are tolerant and productive.

In this sense, studies aiming to provide information on the molecular machinery through which C_4_ plants respond to increased atmospheric CO_2_ are fundamental to supply genetic data to the scientific community. Several gene groups (more than 1300 differentially expressed genes) were identified by Ge et al. [91] through analysis of the maize transcriptome under high [CO_2_]. These data, associated with physiological aspects, can provide additional support to understand the effect of this increase and the interactive effect of stresses.

## 6. Conclusions

The physiological responses of C_4_ plants to increased [CO_2_], such as transpiration rate, stomatal conductance decreases, and photosynthetic enzymes are differentially expressed in maize, sugarcane, and sorghum. The gene regulation involved in carbohydrate metabolism and plant growth changes according to time of exposure to stress and experimental aspects. 

C_4_ plants can present advantages in high [CO_2_] environments and, owing to climatic forecasts involving increased temperature and decreased precipitation, the understanding of adaptive physiological and genetic mechanisms is fundamental in order to guarantee the satisfactory growth of these plants.

## Figures and Tables

**Table 1:** Experimental designs established in some research with C_4_ plants (maize, sugarcane, and sorghum) and elevated atmospheric concentration of carbon dioxide ([CO_2_]) environments.

Plant	[CO_2_] Applied ^1^	Experimental Conditions ^2^	Reference
*Zea mays*	AC: 370 µmol mol^−1^	Soil–plant–atmosphere research chambers	[60]
	EC: 750 µmol mol^−1^		
	AC: 350 µL L^−1^	CEC	[9]
	EC: 700 µL L^−1^		
	AC: 378 µL L^−1^	FACE	[61]
	EC: 550 µL L^−1^		
	AC: 394 ppm	OTCs	[19]
	EC: 566 ppm		
	AC: 400 ppm	OTCs	[62]
	EC: 550 ppm		
*Saccharum* spp.	AC: 360 µmol mol^−1^	TGG	[12]
	EC: 720 µmol mol^−1^		
	AC: 370 ppm	OTCs	[13]
	EC: 720 ppm		
	AC: 360 µmol mol^−1^	TGG	[63]
	EC: 720 µmol mol^−1^		
	AC: 400 ppm	OTCs	[59]
	EC: 750−800 ppm		
*Sorghum bicolor*	AC: 370 µmol mol^−1^	FACE	[64]
	EC: 570 µmol mol^−1^		
	AC: 370 µmol mol^−1^	FACE	[65]
	EC: 570 µmol mol^−1^		
	AC: 400 µmol mol^−1^	OTCs	[66]
	EC: 800 µmol mol^−1^		

^1^ AC = ambient condition; EC = under elevated [CO_2_]. ^2^ CEC = controlled environment cabinets; FACE = free air CO_2_ enrichment; OTCs = open top chambers; TGG = temperature gradient greenhouses.

**Table 2:** Growth responses of C_4_ plants (maize, sugarcane, and sorghum) under elevated [CO_2_] conditions.

Plant	Growth Parameters	Elevated [CO_2_]	Effects *
*Zea mays*	Leaf area	550 ppm	(↑) ^1^ (↑) ^2^
	Plant height	550 ppm	(↑) ^1^ (↑) ^2^
	Total masses	70 Pa–550 ppm	(↑) ^3^ (=) ^4^
*Saccharum* spp.	Leaf area	720 µmol mol^−1^	(↑)^5^
	Plant height	720 ppm	(↑)^6^
	Total masses	720 ppm	(↑)^6^
*Sorghum bicolor*	Leaf area	795–900 µ L^−1^	(↑) ^7^ (↑) ^8^
	Plant height	795 µ L^−1^	(=) ^7^
	Total masses	900 µ L^−1^	(↑) ^8^

* Reference: ^1^ Mina et al. [68]; ^2^ Adishesha et al. [74]; ^3^ Sicher and Barnaby [75]; ^4^ Huang et al. [62]; ^5^ Vu et al. [12]; ^6^ Souza et al. [13]; ^7^ Chaudhuri et al. [76]; ^8^ Khanboluki et al. [69]. The symbols mean an increase (↑) and no difference (=) in relation to control plants ([CO_2_] ambient).
